# Nux Vomica in Parturient Apoplexy

**Published:** 1899-07

**Authors:** E. C. Porter

**Affiliations:** New Castle, Pa.


### NUX VOMICA IN PARTURIENT APOPLEXY.

By Dr. E. C. Porter,
OF NEW CASTLE, PA.

In a paper read at the semi-annual meeting of the Pennsylvania State Veterinary Medical Association, Pittsburg, September, 1898, it was said that a fair measure of success can be obtained by the use of nux vomica fluid extract in the treatment of parturient apoplexy. After placing the patient on sternum, and kept there with bundles of straw so arranged that the patient cannot roll on her side, which will usually alleviate tympanitis or prevent it, then give two drachms fluid extract nux vomica on tongue, which is followed with one-drachm doses every two hours until the toxic effect of the drug is noticed. Then lengthen the period between doses one, two or three hours. Apply some irritant to the back. I usually use ammoniacal liniment well rubbed in two or three times daily. If the body is cold blanket well. Use the catheter and remove fecal matter from rectum twice daily. Patient should be turned every three or four hours from one side to the other. I find the most fatal cases happen when the pasture is rank in the spring, or at any time green feed is used, such as young rye or green corn fodder. The longer the period after calving the more chance of recovery. During the fall of 1896 and spring of 1897 I had ten recoveries out of about fourteen cases; since then I do not think there have been over 50 per cent. recovered.

